# Glucagon-Like Peptide-1 Receptor Agonists and Pancreatic Cancer Risk in Patients With Type 2 Diabetes

**DOI:** 10.1001/jamanetworkopen.2023.50408

**Published:** 2024-01-04

**Authors:** Rachel Dankner, Havi Murad, Nirit Agay, Liraz Olmer, Laurence S. Freedman

**Affiliations:** 1 Public Health Research Center, Gertner Institute for Epidemiology and Health Policy Research, Sheba Medical Center, Ramat Gan, Israel; 2 Department for Epidemiology and Preventive Medicine, School of Public Health, Sackler Faculty of Medicine, Tel Aviv University, Tel Aviv, Israel; 3 Biostatistics and Biomathematics Unit, Gertner Institute for Epidemiology and Health Policy Research, Sheba Medical Center, Ramat Gan, Israel

## Abstract

**Importance:**

Concerns have been raised that glucagon-like peptide-1 receptor agonists (GLP-1RA) may increase the risk of pancreatic cancer.

**Objective:**

To investigate the association of GLP-1RA treatment with pancreatic cancer incidence over 9 years of follow-up.

**Design, Setting, and Participants:**

In this population-based historical cohort study, adult patients (aged 21 to 89 years) with type 2 diabetes insured by Clalit Healthcare Services, the largest state-mandated health organization in Israel, were followed up from 2009, when GLP-1RA became available in Israel, until pancreatic cancer diagnosis, death, reaching age 90 years, or end of follow-up (December 2017). Data were analyzed from June 2022 to November 2023.

**Exposures:**

Treatment with GLP-1RA was compared with basal insulin.

**Main Outcome and Measures:**

Pancreatic cancer incidence was compared according to weighted cumulative exposures to GLP-1RA and to basal insulin in a Cox model implemented in discrete time, with time origin at 2 years after diabetes diagnosis, adjusting for confounding. In sensitivity analyses, propensity score–matched pair new-user design and prevalent new-user design were used for the comparison. Because of risk for reverse-causation bias, results in the fifth to seventh year after medication were emphasized.

**Results:**

During a cumulative follow-up of 3 290 439 person-years of 543 595 adults with a mean (SD) age of 59.9 (12.8) years (277 502 women [51%]) with incident diabetes, 1665 patients received pancreatic cancer diagnoses. In total, 33 377 patients (6.1%) used GLP-1RA and 106 849 (19.7%) used basal insulin. The estimated hazard ratio (HR) for pancreatic cancer associated with incremental use of 1 defined daily dose per day of GLP-1RA compared with basal insulin in the fifth to seventh year previously (all other characteristics, including age, sex, ethnic background, sociodemographic status, baseline body mass index, smoking history, history of pancreatitis, other glucose-lowering medications treatment history, and length of diabetes, being equal) was 0.50 (95% CI, 0.15-1.71). The new-user and prevalent new-user designs showed HRs from the fifth year onwards following initiation of GLP-1RA vs basal insulin of 0.52 (95 % CI, 0.19-1.41) and 0.75 (95 % CI, 0.37-1.53), respectively.

**Conclusions and Relevance:**

In this historical cohort study of adults with type 2 diabetes, no support for an increased pancreatic cancer incidence over 7 years following start of GLP-1RA treatment was found. However, monitoring for pancreatic cancer risk beyond 7 years following initiation of therapy is still required.

**Trial Registration:**

ClinicalTrials.gov Identifier: NCT02072902

## Key Points

**Question:**

Is treatment with glucagon-like peptide-1 receptor agonists (GLP-1RA) of patients with type 2 diabetes associated with excess risk of pancreatic cancer?

**Findings:**

In this cohort study of 543 595 patients, compared with treatment with basal insulin, treatment of comparable patients with type 2 diabetes with GLP-1RA was not associated with excess risk of pancreatic cancer.

**Meaning:**

Using several analytic approaches, these findings do not suggest an increase in pancreatic cancer incidence over 7 years following start of GLP-1RA treatment.

## Teaser

This cohort study evaluates the association of glucagon-like peptide-1 receptor agonist (GLP-1RA) treatment with pancreatic cancer incidence over 9 years of follow-up.

## Introduction

Concerns were raised following several publications that identified an increased risk for pancreatitis and pancreatic cancer in patients taking GLP-1 receptor agonists (GLP-1RA).^1,2^ A Food and Drug Administration (FDA) warning on pancreatic safety followed,^3^ urging both patients and health care professionals to report adverse events involving incretin mimetics to the FDA MedWatch program. Two short-term in vivo studies performed at the FDA’s request^4,5^ and a review of case reports^6^ further increased concerns about the possible adverse effects of GLP-1RA therapy on exocrine pancreas, leading to an elevation in pancreatic enzymes and acute pancreatitis. Nonetheless, more recent meta-analyses,^7,8^ 1 including only trials with adjudicated pancreatitis adverse events,^7^ showed neither an association of GLP-1RA with acute pancreatitis nor with pancreatic cancer. Two large cohort studies^9,10^ published in 2016 also reported negative findings. Three further meta-analyses of randomized clinical trials (RCTs)^11,12^ and observational studies^13^ reported no association between GLP-1RA use and pancreatitis or pancreatic cancer, but an increased risk of cholelithiasis was observed in the first.^11^ Nevertheless, major shortcomings, such as fairly short mean follow-up times (of <2 years in the RCTs and <5 years in the observational studies), limited sample size,^11,12^ and various time-related biases^13^ were noted. Recently, increased risk for pancreatic cancer was described using the FDA Adverse Event Reporting System (FAERS) database for adverse events reported from 2004 to 2020 for GLP-1RA treatment-associated neoplasms, compared with other glucose-lowering medications (GLMs).^14^

We investigated the association of GLP-1RA treatment with pancreatic cancer incidence over a mean (SD) follow-up of 6.1 (2.9) years (median [IQR], 7.0 [3.5-8.8] years) in a population-based historical cohort of more than 500 000 patients with type 2 diabetes (T2D). We used basal insulin as an active comparator while accounting for major confounding factors and time-related biases, as well as adjusting for treatment with other GLMs and history of pancreatitis.

## Methods

The data for this cohort study were obtained from the electronic database of Clalit Healthcare Services (Tel Aviv, Israel), the largest health maintenance organization (HMO) in Israel, insuring over 4 million people, comprising a representative 53% of the total population. The review boards of Sheba Medical Center and Clalit Health Services approved the study protocol. The study investigators were exempted from obtaining informed consent from participants because of the historical nature and source of the data (electronic records on a large population). This paper follows the Strengthening the Reporting of Observational Studies in Epidemiology (STROBE) reporting guideline. Available data include clinical measures, such as body mass index (BMI), blood glucose and glycated hemoglobin (HbA_1c_) levels, smoking history, history of pancreatitis, and sociodemographic information including age, socioeconomic status (SES, determined by locality of the Clalit clinic), and ethnicity (investigator observed and determined by country of birth: Ashkenazi Jewish [born in Eastern Europe, Europe, Russia, South Africa, or the United States]; Central African or Ethiopian Jewish; Israeli Arab; Israeli-born Jewish [when first generation in Israel, the mother’s country of birth determined ethnicity]; Sephardic Jewish [born in the Middle East or North Africa]; or Yemenite Jewish). Ethnicity was analyzed in this cohort study as a potential confounder since it is associated with both the study outcome and the study exposure. Data on dispensation of medications were available since 1998 and included the following GLMs: GLP-1RA, metformin, insulin, α-glucosidase inhibitors, rosiglitazone, sulfonylureas, dipeptidyl peptidase-4 inhibitors, and meglitinides.

For pancreatic cancer diagnosis, the database was linked to the Israel Cancer Registry. Cancer registration is mandated by law in Israel, and the registry reports 97% coverage of solid tumors.^15^

The study population comprised men and women aged 21 to 89 years who received diabetes diagnoses from 2002 onwards (see Figure, A). Diabetes diagnosis was defined as fulfillment of at least 1 of the 6 following criteria: (1) a physician’s diagnosis plus a plasma glucose test result of 126 mg/dL or higher within a 12-month period (to convert to millimoles per liter, multiply by 0.0555); (2) a record of diabetes in the Clalit Chronic Disease Registry; (3) an HbA_1c_ level 6.5% or higher (to convert to proportion of hemoglobin, multiply by 0.01); (4) 2 plasma glucose measurements 126 mg/dL or higher within a 12-month period; (5) a 2-hour plasma glucose concentration after oral glucose tolerance test 200 mg/dL or higher; or (6) 3 or more purchases of GLMs within a 12-month period. The date of diagnosis was defined as the earliest occurrence of 1 of these criteria. Those receiving diabetes diagnoses according to the above criteria before age 30 years, those who received insulin as first-line therapy, and those recorded as having type 1 diabetes diagnoses were excluded.

**Figure.**
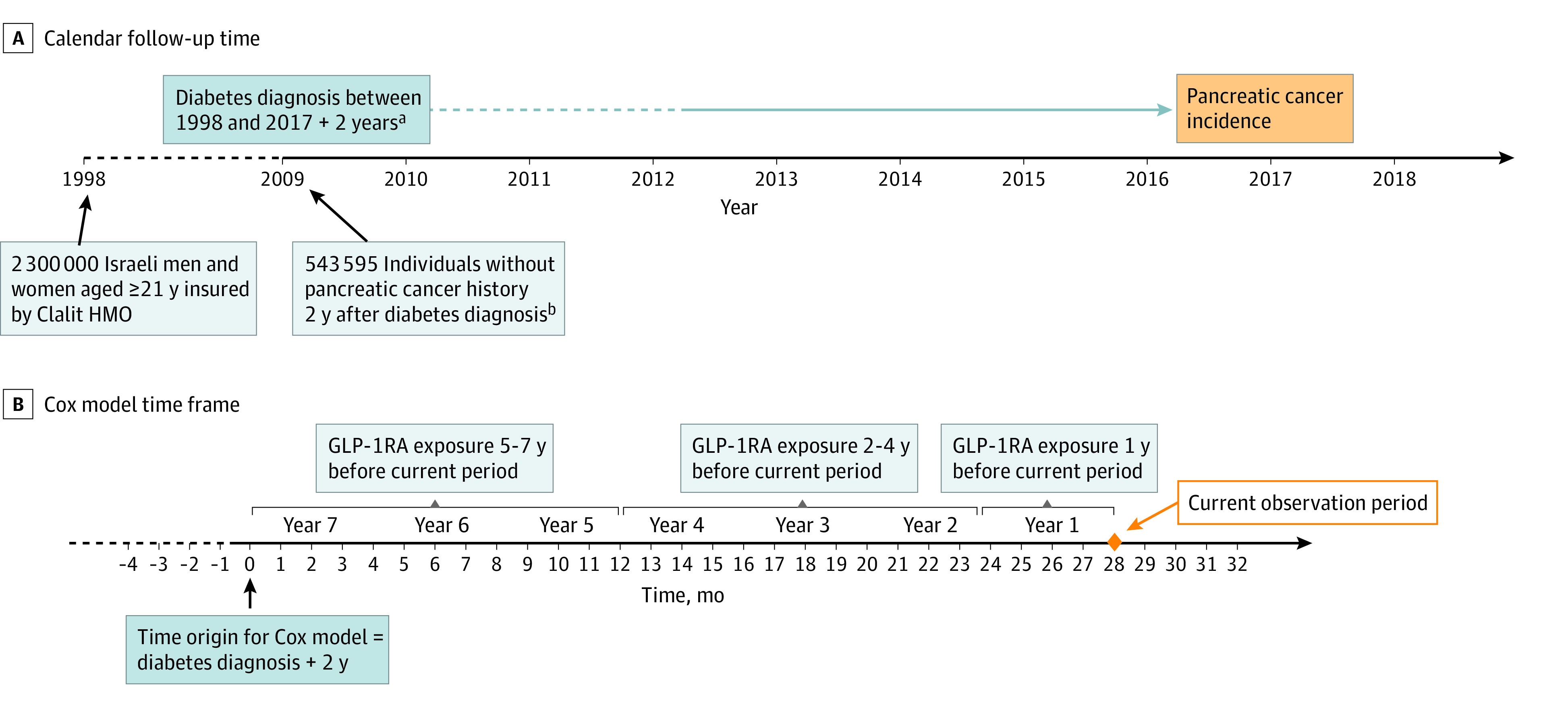
Calendar Follow-Up Time of Individuals Without Pancreatic Cancer at Least 2 Years After Diabetes Diagnosis and Cox Models Time Frame GLP-1RA indicates glucagon-like peptide-1 receptor agonist; HMO, health management organization. ^a^For those diagnosed before 2007, follow-up starts only in 2009. ^b^All Clalit Healthcare Services members who received type 2 diabetes diagnoses from 1998 to 2017 and were free of pancreatic cancer by January 1, 2009.

### Statistical Analysis

We evaluated pancreatic cancer risk associated with GLP-1RA use by comparing it with pancreatic cancer risk associated with use of basal insulin. Both medications were used as injectable second-line therapy for type 2 diabetes.

Unadjusted comparisons of the baseline characteristics by GLP-1RA ever-users and basal insulin ever-users were made using the χ^2^ test for discrete variables, and the unpaired *t* test for continuous variables. Crude annual rates of pancreatic cancer among ever-users of GLP-1RA and basal insulin were computed.

To first check on whether history of pancreatitis was associated with GLP-1RA prescription, a Cox model for the time until starting GLP-1RA treatment was built with pancreatitis as a time-dependent covariate, equal to 0 before pancreatitis diagnosis and equal to 1 thereafter. The model was adjusted for age, sex, ethnic origin, SES, and BMI at baseline, and follow-up started at diabetes diagnosis or beginning of 2009 (the year when GLP-1RA became available in Israel), whichever was later, and ended at GLP-1RA first prescription, death, age 90 years, or end of 2017. A similar model was built for the time until starting basal insulin treatment (see eAppendix 1 in Supplement 1 for further details). Because substantial associations were seen in these analyses, adjustment for pancreatitis was made in our analyses (see below).

The main analyses compared the incidence of pancreatic cancer associated with GLP-1RA use with that associated with basal insulin use. The comparisons were based upon discrete-time Cox models with time-dependent covariates, using 3-month periods (the interval between GLM prescriptions) with time origin at 2 years post–diabetes diagnosis and continuing until end of follow-up (pancreatic cancer diagnosis, death, reaching age 90 years, or December 31, 2017). We started follow-up 2 years post–diabetes diagnosis to avoid the problem of reverse causation bias, whereby undiagnosed pancreatic cancer causes symptoms leading to diagnosis of diabetes before the cancer itself is diagnosed. The exposures (GLP-1RA and basal insulin) were modeled as weighted cumulative exposures through time,^16^ as described in Dankner et al^17^ (see eAppendix 2 in Supplement 1). Average GLP-1RA and basal insulin uses (in units of defined daily dose [DDD]) were computed over 3 nonoverlapping time windows: the first year, the second to fourth years, and the fifth to seventh years before the current 3-month period. The hazard ratios (HR) per 1 DDD exposure for each period of use were estimated for the 2 medications, and their ratio was computed as the HR for GLP-1RA vs basal insulin.

As we included follow-up information from 2009 onwards (the year GLP-1RA first became available in Israel), patients who received diabetes diagnoses before 2007 entered follow-up later than at the time origin (2 years after diabetes diagnosis). To accommodate this delayed entry, we followed the method of Lamarca et al.^18^ For details, see eAppendix 3 and eFigure 1 in Supplement 1.

Since physicians selectively prescribe GLP-1RA or basal insulin to patients with specific characteristics, introducing selection bias in the GLP-1RA and basal insulin treated groups, we adjusted the models for several potentially important confounders. We adjusted for history of pancreatitis (as a time-dependent covariate) diagnosed before taking the specified medications but not pancreatitis occurring after initiation of GLP-1RA or basal insulin, since the latter is not a confounder. Other confounder variables adjusted for included age in 5 groups (21-50, 51-60, 61-70, 71-80, and 81-90 years), sex, ethnicity, sociodemographic status (low, medium, high, or missing ), smoking history (never or missing, past, and current), BMI at baseline (<25, 25-29.9, ≥30, or missing; calculated as weight in kilograms divided by height in meters squared), and history of other GLMs using the same 3 time windows as used with GLP-1RA and basal insulin. Defining time since diabetes diagnosis as the time scale (Figure, B) automatically adjusted for any confounding due to length of diabetes.

In our analyses, we also adjusted for potential bias due to another type of reverse-causation, whereby yet undiagnosed pancreatic cancer may cause disturbance of glucose metabolism, leading to higher glucose and HbA_1c_ levels, thus inducing prescription of GLP-1RA or basal insulin as an intensification second-line treatment. If present, this phenomenon is expected to operate during the year preceding diagnosis of pancreatic cancer, and possibly in the second to fourth years back.^19^ To mitigate this effect, we conducted analyses that excluded from the Cox models the variables for GLM treatments in the previous year (see eFigure 2 in Supplement 1) or both the previous year and the second to fourth years back.

Sensitivity analyses using a propensity score–matched new-user design and prevalent new-user design^20^ were also conducted to compare pancreatic cancer risk among GLP-1RA users vs basal insulin users. The methods are described in eAppendix 4 in Supplement 1.

All analyses were conducted with SAS version 9.4 (SAS Institute). Data were analyzed from June 2022 to November 2023.

## Results

In this study, 543 595 individuals (277 502 (51%) women) with diabetes were followed up over a 9-year period (mean [SD] 6.1 [2.9] years), with 3 290 439 person-years with diabetes accrued. Table 1 presents the baseline characteristics of the cohort according to use of GLP-1RA and basal insulin. The mean (SD) age of the cohort was 59.9 (12.8) years, and they predominantly had overweight (37% with BMI 25-29.9) or obesity (42% with BMI ≥30). The majority (320 713 participants [59.0%]) were of low SES, and 194 861 (35.8%) had a positive smoking history.

**Table 1.** Baseline Characteristics of 543 595 Israeli Individuals With Incident Diabetes, Followed Up From 2009 to 2017 for Pancreatic Cancer Incidence (3 290 439 Person-Years) According to Glucagon-Like Peptide-1 Receptor Agonist (GLP-1RA) Ever-Users and Basal Insulin Ever-Users

Characteristic	Participants, No. (%)		
	GLP-1RA users (n = 33 386)^a^^,^^b^	Basal insulin users (n = 106 849)^a^^,^^b^	Full cohort (N = 543 595)
Age at diabetes diagnosis, mean (SD), y	49.2 (8.8)	54.0 (11.3)	59.9 (12.8)
Sex			
Male	15 495 (46.4)	54 903 (51.4)	266 093 (49.0)
Female	17 891 (53.6)	51 946 (48.6)	277 502 (51.0)
BMI^c^			
<25	679 (2.0)	17 156 (16.1)	102 768 (18.9)
25-29.9	6787 (20.3)	37 452 (35.1)	200 743 (36.9)
≥30	25 755 (77.2)	50 860 (47.6)	226 794 (41.7)
Missing	165 (0.5)	1381 (1.3)	13 290 (2.4)
Ethnic origin			
Ashkenazi Jewish	8766 (26.3)	25 901 (24.2)	168 345 (31.0)
Ethiopian and Central African Jewish	201 (0.6)	1462 (1.4)	7369 (1.4)
Israeli Arab	9547 (28.6)	29 900 (28.0)	98 387 (18.1)
Israeli-born Jewish	6923 (20.7)	18 444 (17.3)	100 026 (18.4)
Sephardic Jewish	7169 (21.4)	26 817 (25.1)	148 954 (27.4)
Yemenite Jewish	780 (2.3)	4325 (4.1)	20 514 (3.8)
Socioeconomic status			
Low	20 971 (62.7)	70 069 (66.2)	320 713 (59.0)
Medium	11 371 (34.1)	33 185 (31.1)	204 012 (37.5)
High	1010 (3.0)	2677 (2.5)	17 280 (3.2)
Missing	34 (0.1)	281 (0.2)	1590 (0.3)
Smoking			
Never smoker	20 376 (61.1)	64 378 (60.3)	339 157 (62.4)
Past smoker	4397 (13.2)	13 340 (12.5)	68 190 (12.5)
Current smoker	8601 (25.7)	28 351 (26.5)	126 671 (23.3)
Missing	12 (0.03)	780 (0.7)	9577 (1.8)
History of pancreatitis	1090 (3.3)^d^	3714 (3.5)^e^	13 783 (2.5)

^a^ Patients were prescribed GLP-1RA or basal insulin in at least 1 period (3 months) during their follow-up.

^b^ There is overlap between the GLP-1RA and basal insulin ever-users groups; approximately half of those taking GLP-1RA also took basal insulin.

^c^ Body mass index is calculated as weight in kilograms divided by height in meters squared.

^d^ A total of 480 (1.4%) received pancreatitis diagnoses before starting treatment with GLP-1RA.

^e^ A total of 1756 (1.6%) received pancreatitis diagnoses before starting treatment with basal insulin.

GLP-1RA ever-users (33 386 individuals [6.1%]) were on average younger (*t*_14 314_ = 48.1) with a greater proportion of obesity (χ^2^_3_ = 6433) than basal insulin ever-users (106 849 individuals [19.7%]). These differences were statistically significant at *P* < .001.

Pancreatitis had a similar prevalence among GLP-1RA and basal insulin users (3.3% vs 3.5%, although χ^2^_1_ = 114.9 and *P* < .001 due to large numbers) (Table 1). However, this analysis did not consider the timing of the pancreatitis covariate, nor the differences in other characteristics. When exploring the association between previous pancreatitis and first treatment with GLP-1RA or basal insulin using Cox modeling and adjusting for these characteristics, a negative association was found with GLP-1RA (HR, 0.52; 95% CI, 0.48-0.57), and a positive association with basal insulin (HR, 1.36; 95% CI, 1.30-1.42) (see eTable 1 in Supplement 1).

Over the 9-year follow-up period, 1665 patients in the full cohort received pancreatic cancer diagnoses. Compared with noncases, pancreatic cancer cases were on average 4 years older, more likely to be Ashkenazi Jewish patients, were less likely to be Israeli Arab patients, had lower BMI, and were less likely to be in the low SES category.

GLP-1RA use, basal insulin use, and crude annual pancreatic cancer incidence rates are presented by years since diabetes diagnosis in Table 2. As GLP-1RA and basal insulin are not first-line therapies, the proportion of users gradually increased with time since diabetes diagnosis. The crude annual rates of pancreatic cancer were similar among GLP-1RA users and nonusers, but higher among basal insulin users, most probably due to their older age and longer diabetes duration. The results from the Cox models for GLP-1RA and basal insulin are shown in eTable 2 in Supplement 1 and lead to the comparison of GLP-1RA with basal insulin shown in Table 3.

**Table 2.** Person-Years Use and Pancreatic Cancer Incidence According to Glucagon-Like Peptide-1 Receptor Agonist (GLP-1RA) or Basal Insulin Use by Year Since Diabetes Diagnosis, Among 543 595 Individuals With Incident Diabetes^a^

Years since diabetes diagnosis	GLP-1RA				Basal insulin			
	No. of person-years at risk^b^		Pancreatic cancer cases, No. (crude annual rate per 10 000)		No. of person-years at risk^b^		Pancreatic cancer cases, No. (crude annual rate per 10 000)	
	Ever-users^c^	Nonusers	Ever-users	Nonusers	Ever-users^c^	Nonusers	Ever-users	Nonusers	
2-3	1998	490 025	2 (10.0)	191 (3.9)	8830	483 193	25 (28.3)	168 (3.5)	
4-5	4923	495 797	1 (2.0)	212 (4.3)	17 053	483 667	37 (21.7)	176 (3.6)	
6-7	8524	506 516	2 (2.3)	235 (4.6)	29 053	485 987	48 (16.5)	189 (3.9)	
8-9	12 939	481 330	7 (5.4)	250 (5.2)	46 294	447 974	72 (15.6)	185 (4.1)	
10-11	17 372	438 255	11 (6.3)	213 (4.9)	72 517	383 110	70 (9.7)	154 (4.0)	
12-13	21 423	367 510	8 (3.7)	229 (6.2)	98 140	290 793	92 (9.4)	145 (5.0)	
14-15	23 086	239 309	16 (6.9)	159 (6.6)	91 699	170 695	108 (11.8)	67 (3.9)	
16-17	16 363	118 003	11 (6.7)	76 (6.4)	64 637	69 730	56 (8.7)	31 (4.4)	
18-19	11 350	60 203	6 (5.3)	36 (6.0)	40 923	30 630	32 (7.8)	10 (3.3)	
Total	NA	NA	64	1601	NA	NA	540	1125	

Abbreviation: NA, not applicable.

^a^ To calculate the approximate number of persons taking the medications during each period, divide the number of person-years by 2.

^b^ Alive, free of pancreatic cancer, and aged younger 90 years. Number of person-years in each 2-year period, calculated under the assumption that in each cell, all the people completed the whole 2 years.

^c^ Patients prescribed GLP-1RA or basal insulin for at least 1 quarter (3 months) during their follow-up.

**Table 3.** Hazard Ratios (HRs) for the Association of Pancreatic Cancer With 1 Defined Daily Dose (DDD) Increment in Glucagon-Like Peptide-1 Receptor Agonist (GLP-1RA) Use vs 1 DDD Increment in Basal Insulin Use During the 7 Years Before Cancer Diagnosis^a^

Model	HR (95% CI) for GLP-1RA vs basal insulin, years before pancreatic cancer diagnosis		
	Previous y	Second to fourth y back	Fifth to seventh y back
Full model	0.22 (0.11-0.41)	1.54 (0.51-4.69)	1.06 (0.26-4.33)
Omitting previous year	NA	0.32 (0.13-0.76)	1.43 (0.35-5.79)
Omitting previous year and second to fourth year back	NA	NA	0.50 (0.15-1.71)

Abbreviation: NA, not applicable.

^a^ Adjusted for all other GLMs: metformin, short-acting insulin, α-glucosidase inhibitors, rosiglitazone, sulfonylureas, dipeptidyl peptidase-4 inhibitors or GLP-1RA, meglitinides; and confounding variables: age, sex, socioeconomic status, ethnic origin, smoking, baseline BMI, pancreatitis that occurred before the start of treatment (GLP-1RA/basal insulin). Excludes those who completed follow-up within 2 years of their diabetes diagnosis.

The estimated HR for pancreatic cancer comparing 1 DDD of GLP-1RA vs 1 DDD of basal insulin, adjusted for length of diabetes, age, sex, socioeconomic status, ethnic origin, smoking, baseline BMI, and other GLM use, was 0.22 (95% CI, 0.11-0.41) for medication taken during the previous year. For the periods further back, the HRs were greater than 1 (1.54; 95% CI, 0.51-4.69, and 1.06; 95% CI, 0.26-4.33, respectively), but the 95% CIs covered the null value of 1 (Table 3). Because these results suggested a greater degree of reverse causation for basal insulin than for GLP-1RA, thus leading to a biased comparison,^19^ we conducted further analyses.

Omitting from the Cox models the variables for medication use during the previous year to adjust for reverse-causation during this period, the estimated adjusted HRs associated with GLP-1RA vs basal insulin were 0.32 (95% CI, 0.13-0.76) for medication taken during the second to fourth year previously, and 1.43 (95% CI, 0.35-5.79) for medication taken during the fifth to seventh year previously. When medication during both the previous year and the second through fourth year previously was omitted, the estimated adjusted HR associated with GLP-1RA vs basal insulin was 0.50 (95% CI, 0.15-1.71) (Table 3). Thus, all CIs either fell below or covered the null value 1.

In the new-user design, HRs for pancreatic cancer in GLP-1RA users vs basal insulin users were 0.67 (95% CI, 0.41-1.07) over the full follow-up period, 0.84 (95% CI, 0.46-1.52) omitting the first year of follow-up, and 0.52 (95% CI, 0.19-1.41) omitting the first 4 years of follow-up (eTable 3 in Supplement 1). In the prevalent new-user design, HRs for pancreatic cancer in GLP-1RA users vs basal insulin users were 1.03 (95% CI, 0.72-1.47) over the full follow-up period, 1.13 (95% CI, 0.73-1.76) omitting the first year of follow-up, and 0.75 (95% CI, 0.37-1.53) omitting the first 4 years of follow-up (eTable 3 in Supplement 1). See eAppendix 4 and eTable 4 in Supplement 1 for further details of these analyses.

## Discussion

Our analyses of a large cohort of individuals with newly diagnosed diabetes over a 9-year follow-up period with more than 33 000 GLP-1RA users and a fairly large number of incident pancreatic cancers seem not to support an increase in pancreatic cancer risk associated with GLP-1RA use. Other publications are in line with our findings. In a nested case-control study^9^ of median follow-up ranging from 1.3 to 2.8 years with 274 pancreatic cancer incidents, compared with sulfonylureas, GLP-1RA use was not associated with an increased risk of pancreatic cancer (adjusted HR, 1.13; 95% CI, 0.38-3.38).

In a systematic review^11^ that included 113 trials on the safety of GLP-1RAs, the incidence of pancreatitis and pancreatic cancer with GLP-1RA was not significantly different from that observed in the comparator groups (odds ratio [OR], 0.93; 95% CI, 0.65-1.34, and 0.94; 95% CI, 0.52-1.70, respectively). Nevertheless, a significantly increased risk of cholelithiasis (OR, 1.30; 95% CI, 1.01-1.68) was detected.

A systematic review with meta-analysis published in 2018 included 4 major GLP-1RA cardiovascular safety trials with adjudication for several safety signals including pancreatitis and pancreatic cancer,^8^ and did not support an increase in pancreatitis (OR, 0.90; 95% CI, 0.63-1.28) or pancreatic cancer (HR, 0.83; 95% CI, 0.33-2.11) associated with GLP-1RA treatment compared with placebo.

In another systematic review with meta-analysis^12^ of randomized trials with GLP-1RA as an intervention, 12 trials were included with a total of 36 397 patients. GLP-1RA did not appear to increase the risk for pancreatic cancer when compared with other treatments (OR, 1.06; 95% CI, 0.67-1.67; *I*^2^ = 14%).

Seven trials with a total of 56 000 participants were included in a 2019 systematic review with meta-analysis that did not find a difference between GLP-1RA treatment and placebo in the incidence of pancreatitis or pancreatic cancer.^21^ Nevertheless, a recently published pharmacovigilance study^14^ of the FDA FAERS data for malignant neoplasm–related reporting of adverse events spanning from 2004 to 2020 found an almost 10 times greater disproportional reporting ratio for pancreatic cancer related to GLP-1RA treatment as compared with other GLMs.

### Strengths and Limitations

The current study has strengths. The Clalit database is known to be of high quality, and has been the source of many research reports^17,22,23,24,25^ assuring the high validity of our findings. Our analysis was adjusted for potentially important factors associated with risk for pancreatic cancer, such as smoking, obesity, and history of pancreatitis before GLP-1RA or basal insulin treatment. As noted in the Methods section and confirmed in our Results section, physicians may avoid prescribing GLP-1RA to patients with a history of pancreatitis or high amylase, potentially introducing differential selection bias in the GLP-1RA treated patient population.

To study the association of GLP1-RA use with pancreatic cancer incidence, we used a Cox regression model with time varying covariates, an analysis that is free from the typical biases caused by restricting the study population according to strict inclusion criteria and that provides excellent external validity. The time origin for the Cox model was chosen as 2 years after diabetes diagnosis. Using time since diabetes diagnosis for the time axis controls for any confounding due to length of disease and avoids any immortal time bias in defining the GLP-1RA user group. Starting at 2 years after diagnosis minimizes inclusion of cases of pancreatic cancer that led to diabetes diagnosis while yet undetected themselves (ie, mitigating bias due to 1 type of reverse causation). Our analyses, where we omit from the model GLP-1RA exposure in the previous year or the previous 4 years, mitigates bias due to another type of reverse causation, whereby undiagnosed pancreatic cancer disturbs glucose metabolism and leads to prescription of GLP-1RA, perhaps as second-line treatment. Comparing GLP-1RA with basal insulin, a GLM class recently observed not to be associated with pancreatic cancer risk,^26^ ensures comparison of patients who require second-line therapy and avoids their comparison with patients requiring less intensive therapy for their diabetes. Inclusion of multiple methods of analysis that lead to similar results strengthens our conclusions.

This study also has limitations. Because GLP-1RA became available in Israel only in 2009, we included follow-up information only from that year onwards, thus avoiding biases due to different follow-up periods for exposed and nonexposed patients. Exact type of GLP-1RA was not available in our data.

Because of the risk of bias due to reverse causation, we emphasize analyses of drug effects several years after their initiation. However, this reduces the number of pancreatic cancer cases available for these analyses and leads to estimated HRs with wider CIs.

We did not adjust the models for HbA_1c_ and glucose levels because their levels may be affected by reverse causation (see the causal pathway diagram, eFigure 2 in Supplement 2), so their inclusion in the model would bias the estimation of the associations between GLP-1RA or basal insulin and pancreatic cancer incidence. Although we were able to adjust the analyses to history of pancreatitis, we could not account for alcohol use or past exposure to pesticides or chemicals. Additionally, although our definition of diabetes may have allowed inclusion of both type 1 and type 2 diabetes, the proportion of patients older than 30 years receiving insulin as their first treatment was 1.8%, indicating that over 98% of patients included in the analysis had type 2 diabetes.

## Conclusions

In summary, in this historical cohort of more than half a million adults with diabetes, we did not reveal any compelling evidence of increased pancreatic cancer risk following use of GLP-1RA. We were able to follow more than 30 000 GLP-1RA users and evaluate their risk for pancreatic cancer incidence up to 7 years after initiation. Our Cox model allowed us to explore the risk for pancreatic cancer associated with use of GLP-1RA compared with basal insulin, with all other characteristics being equal, including history of pancreatitis, other glucose-lowering drugs, and length of diabetes. Our new-user and prevalent new-user design analyses, comparing GLP-1RA use with basal insulin use, concurred with these findings. However, monitoring of GLP-1RA for pancreatic cancer risk beyond 7 years following initiation of therapy is still required.

## Notes

Supplement 1. **eAppendix 1. **Cox Regression Model for the Time Until GLP1-RA or Basal Insulin Treatment **eAppendix 2. **Cox Model for Relating GLP-1RA and Basal Insulin Medication to Pancreatic Cancer Incidence **eAppendix 3. **Delayed Entry Model According to Lamarca et al **eFigure 1. **Examples of Delayed Entry in 4 Individuals **eFigure 2. **Directed Acyclic Graph (DAG) for the Causal Association Between Specified GLM (either GLP1-RA or Basal Insulin) and Pancreatic Cancer **eAppendix 4. **New-User and Prevalent New-User Designs **eTable 1. **Cox Models for Factors Associated With Starting GLP1-RA Treatment (N=553 544; 33 749 GLP1-RA Initiation Events), and for Starting Basal Insulin Treatment (N=687 437; 108 194 Basal Insulin Initiation Events) **eTable 2. **Results From a Cox Model for the Association of Pancreatic Cancer With GLP-1RA and Basal Insulin Exposures (DDD) in the Previous 7 Years **eTable 3. **Baseline Characteristics by Treatment Group for the Incident New-Users and Prevalent New-Users Designs **eTable 4. **Estimated Hazard Ratios for the Association of GLP-1RA Exposure vs Basal Insulin Exposure With Pancreatic Cancer
eReferences.

Supplement 2.
Data Sharing Statement
