# Supplementary material for: Glucagon-Like Peptide-1 Receptor Agonists and Pancreatic Cancer Risk in Patients With Type 2 Diabetes
Source: JAMA Netw Open. 2024 Jan 4;7(1):e2350408. doi: 10.1001/jamanetworkopen.2023.50408 (PMC10767614; doi:10.1001/jamanetworkopen.2023.50408)
Supplement: Supplement 1. — eAppendix 1. Cox Regression Model for the Time Until GLP1-RA or Basal Insulin Treatment eAppendix 2. Cox Model for Relating GLP-1RA and Basal Insulin Medication to Pancreatic Cancer Incidence eAppendix 3. Delayed Entry Model According to Lamarca et al eFigure 1. Examples of Delayed Entry in 4 Individuals eFigure 2. Directed Acyclic Graph (DAG) for the Causal Association Between Specified GLM (either GLP1-RA or Basal Insulin) and Pancreatic Cancer eAppendix 4. New-User and Prevalent New-User Designs eTable 1. Cox Models for Factors Associated With Starting GLP1-RA Treatment (N=553 544; 33 749 GLP1-RA Initiation Events), and for Starting Basal Insulin Treatment (N=687 437; 108 194 Basal Insulin Initiation Events) eTable 2. Results From a Cox Model for the Association of Pancreatic Cancer With GLP-1RA and Basal Insulin Exposures (DDD) in the Previous 7 Years eTable 3. Baseline Characteristics by Treatment Group for the Incident New-Users and Prevalent New-Users Designs eTable 4. Estimated Hazard Ratios for the Association of GLP-1RA Exposure vs Basal Insulin Exposure With Pancreatic Cancer eReferences. [file jamanetwopen-e2350408-s001.pdf]

Supplemental Online Content

Dankner R, Murad H, Agay N, Olmer L, Freedman LS. GLP-1 receptor agonist and pancreatic cancer risk in patients with type 2 diabetes. *JAMA Netw Open*. 2024;7(1):e2350408. doi:10.1001/jamanetworkopen.2023.50408

eAppendix 1. Cox Regression Model for the Time Until GLP1-RA or Basal Insulin Treatment

eAppendix 2. Cox Model for Relating GLP-1RA and Basal Insulin Medication to Pancreatic Cancer Incidence

eAppendix 3. Delayed Entry Model According to Lamarca et al

eFigure 1. Examples of Delayed Entry in 4 Individuals

eFigure 2. Directed Acyclic Graph (DAG) for the Causal Association Between Specified GLM (either GLP1-RA or Basal Insulin) and Pancreatic Cancer

eAppendix 4. New-User and Prevalent New-User Designs

eTable 1. Cox Models for Factors Associated With Starting GLP1-RA Treatment (N=553 544; 33 749 GLP1-RA Initiation Events), and for Starting Basal Insulin Treatment (N=687 437; 108 194 Basal Insulin Initiation Events)

eTable 2. Results From a Cox Model for the Association of Pancreatic Cancer With GLP-1RA and Basal Insulin Exposures (DDD) in the Previous 7 Years

eTable 3. Baseline Characteristics by Treatment Group for the Incident New-Users and Prevalent New-Users Design

eTable 4. Estimated Hazard Ratios for the Association of GLP-1RA Exposure vs Basal Insulin Exposure With Pancreatic Cancer

eReferences.

This supplemental material has been provided by the authors to give readers additional information about their work.

© 2024 Dankner R et al. *JAMA Network Open.*

eAppendix 1. Cox Regression Model for the Time Until GLP1-RA or Basal Insulin Treatment

In order to examine the association between pancreatitis and treatment with each of the medications, GLP-1RA and basal insulin, we built two Cox regression models: one for the time until starting GLP-1RA, and the other, for the time until starting basal insulin treatment. The origin for the time axis was the date of diabetes diagnosis. Since GLP-1RA entered the Israeli health system only in 2009, we used the delayed entry method[1] (see eSupplement – part 3) for that model, in order to include patients who were diagnosed with diabetes before 2009, without introducing immortal-time bias. According to this method, follow up started at diabetes diagnosis (or for GLP-1RA at the beginning of 2009, whichever was later). Follow up ended at first relevant prescription (GLP-1RA or basal insulin), death, age 90 or end of 2017, whichever came first. Pancreatitis was modeled as a time-dependent covariate, equal to zero before pancreatitis incidence and equal to one thereafter. The model was adjusted for age, sex, ethnic origin, SES and BMI at baseline.

eTable 1. Cox Models for Factors Associated With Starting GLP1-RA Treatment (N=553 544; 33 749 GLP1-RA Initiation Events), and for Starting Basal Insulin Treatment (N=687 437; 108 194 Basal Insulin Initiation Events)

**Parameter** (reference)	**Category**	**GLP-1RA Hazard Ratio (95% CI)**	**Basal insulin Hazard Ratio (95% CI)**
**Age group at entry, years** (21-49)	(50-60]	0.57 (0.56, 0.58)	0.70 (0.69, 0.71)
	(60-70]	0.18 (0.17, 0.19)	0.55 (0.54, 0.56)
	(70-80]	0.03 (0.03, 0.04)	0.40 (0.39, 0.41)
	(80-90)	0.01 (0.01, 0.02)	0.50 (0.48, 0.52)
**SES** (Low)	High	1.15 (1.08, 1.22)	0.83 (0.80, 0.86)
	Medium	1.12 (1.09, 1.14)	0.86 (0.85, 0.87)
**Ethnic origin** (Ashkenazi Jewish)	Ethiopian and Central African Jewish	0.61 (0.53, 0.70)	1.23 (1.17, 1.30)
	Israeli Arab	0.98 (0.95, 1.01)	1.67 (1.64, 1.70)
	Israeli Born Jewish	0.94 (0.91, 0.97)	1.04 (1.02, 1.06)
	Sephardic Jewish	0.82 (0.80, 0.85)	1.06 (1.04 1.08)
	Yemenite Jewish	0.88 (0.81, 0.94)	1.30 (1.26, 1.34)
**Sex** (male)	Female	1.01 (0.99, 1.03)	0.91 (0.90, 0.92)
**BMI at entry, Kg/m^2** (below 25)	≥30	12.88 (11.95, 13.89)	1.21 (1.16, 1.25)
	[25,30)	4.63 (4.28, 5.00)	1.03 (0.99, 1.07)
	Missing	1.89 (1.62, 2.20)	2.10 (2.03, 2.17)
**Past pancreatitis** (no)	Yes	0.52 (0.48, 0.57)	1.36 (1.30, 1.42)

© 2024 Dankner R et al. *JAMA Network Open.*

eAppendix 2. Cox Model for Relating GLP-1RA and Basal Insulin Medication to Pancreatic Cancer Incidence

The model used follows the same form as that in Dankner et al (2019)[2]. We denote time since start of follow-up (2 years following diabetes diagnosis), measured in quarters (units of 3-months), by t. We denote drug exposure to the specific drug of interest in period t by $D(t)$, confounding variables by C, and the outcome (i.e., pancreatic cancer or not) in quarter t by $y(t)$, equal to 1 if diagnosed in this period, and to 0 if not yet diagnosed. We denote the hazard rate for the outcome at time t by $\lambda(t)$.

A usual Cox regression model would specify $\lambda(t) = \lambda_0(t)\exp(\beta_1 D(t) + \beta_C C)$, where $\lambda_0(t)$ is the baseline hazard function; however, this model postulates an immediate effect of the current dose of the drug on the risk of the outcome, and no effect of drug taken in the past. Instead, we specified the weighted cumulative model used in our analysis of the pancreatic cancer data as:

$$\lambda(t) = \lambda_0(t)\exp\{\beta_1 D_1(t) + \beta_2 D_2(t) + \beta_3 D_3(t) + \beta_C C\}, \quad (1)$$

where $D_1(t)$, $D_2(t)$ and $D_3(t)$ were the mean daily doses of the drug of interest (in DDD units) over the year previous to quarter t, years 2-4 previous to t and years 5-7 previous to t respectively, for t = 1 onwards. These variables allowed exploration of the association of the drug of interest taken in the short, medium or longer-term past, with pancreatic cancer incidence. The vector C included the confounding covariates mentioned in the main text of the paper.

To accommodate the estimation in the same model of the effects of GLP-1RA and of basal insulin, we expanded model (1) as follows:

$$\lambda(t) = \lambda_0(t)\exp\{\beta_{G1} D_{G1}(t) + \beta_{G2} D_{G2}(t) + \beta_{G3} D_{G3}(t) + \beta_{B1} D_{B1}(t) + \beta_{B2} D_{B2}(t) + \beta_{B3} D_{B3}(t) + \beta_C C\} \quad (2),$$

where the β and D terms with subscript G refer to GLP-1RA and those with subscript B refer to basal insulin. The exponent of the estimated β parameters of this model are shown in eTable 2 below.

eTable 2. Results from a Cox Model[a] for the Association of Pancreatic Cancer With GLP-1RA and Basal Insulin Exposures (DDD) in the Previous 7 Years

**Model**	HR (95% CI) for 1 DDD increment of drug					
	GLP-1RA in previous year	GLP-1RA 2nd-4th year back	GLP-1RA 5th-7th year back	Basal insulin in previous year	Basal insulin 2nd-4th year back	Basal insulin 5th-7th year back
Full model	0.58 (0.32,1.05)	0.65 (0.24,1.79)	0.54 (0.14,2.05)	2.69 (2.09,3.46)	0.42 (0.27,0.66)	0.51 (0.32,0.50)
Omitting previous year	-	0.44 (0.20,0.95)	0.59 (0.16,2.22)	-	1.37 (0.98,1.89)	0.41 (0.26,0.65)
Omitting previous year and 2nd-4th year back	-	-	0.25 (0.08,0.81)	-	-	0.51 (0.37,0.70)

Abbreviations: DDD, defined daily dose; CI, confidence interval; HR, hazard ratio.
[a] Adjusted for: all other GLMs: metformin, insulin, α-glucosidase inhibitors, rosiglitazone, sulfonylureas, DPP-4 inhibitors, meglitinides; Confounding variables: age, sex, socioeconomic status, ethnic origin, smoking, baseline BMI, pancreatitis which occurred before the start of specified GLM (either GLP-1RA or basal insulin); excluding those who completed follow-up within two years of their diabetes diagnosis.

To compare the effect of the new treatment GLP-1RA on the incidence of pancreatic cancer versus the effect of the comparator treatment basal insulin, the difference between the effects of GLP-1RA and the effect of basal insulin for each time-window (i.e., $\beta_{G1} - \beta_{B1}, \beta_{G2} - \beta_{B2}$, and $\beta_{G3} - \beta_{B3}$) and their 95%

© 2024 Dankner R et al. *JAMA Network Open.*

confidence intervals were calculated. The exponent of these terms gives the hazard ratios and their confidence intervals that are shown in Table 3 of the main paper.

© 2024 Dankner R et al. *JAMA Network Open.*

eAppendix 3. Delayed Entry Model According to Lamarca et al (1998)[1]

Starting from the 1st quarter of 2002 until the last quarter of 2017, let the quarters be numbered as *j*=1,2,3, …. ,64.

In our data there is a delayed entry into the risk set since GLP-1RA became available in Israel only in quarter 31 (the 3rd quarter of 2009).

We adopt the following notations:

D is the quarter in which the subject is diagnosed with diabetes,

The time scale origin for the Cox analysis is D+8 (2 years after diabetes diagnosis).

The quarter in which the diabetic patient enters the risk set is then max{31 ,(D+8)}.

W = delay in entry = number of quarters since D+8 until quarter 31 (=31-(D+8)) when D+8<31 and = 0 otherwise).

F is the last quarter of follow-up (pancreatic cancer, death or reaching the age of 90),

if F <= D+8 or F < 31, then the patient does not enter the analysis.

δ (=0 or 1) is the indicator for the event (pancreatic cancer).

Y = number of quarters since entering the risk set until end of follow-up (= F-max{31 ,(D+8)}+1)

T=total number of quarters since D+8 until end of follow-up (W+Y)

We apply the Cox model with the counting process formulation[3,4], which fits the delayed entry problem. The interval of time considered in the analysis is W, where subjects enter the risk set and we start counting events, until T.

In SAS we use the following code for the time dependent average exposure of GLP-1RA in previous year. The covariate glp1_m is time-dependent and therefore it is defined in the programming statement of the PHREG procedure[5]. There is a maximum of 34 quarters in the follow-up (max(Y)=34 , from *j*=31 till *j*=64)

```
Proc Phreg;
model (w,t)*delta(0)= glp1;
array glp1_m[34] glp1_m1-glp1_m34;
glp1=glp1_m[y];
run;
```

Following are a few examples.

© 2024 Dankner R et al. *JAMA Network Open.*

eFigure 1. Examples of Delayed Entry in 4 Individuals*

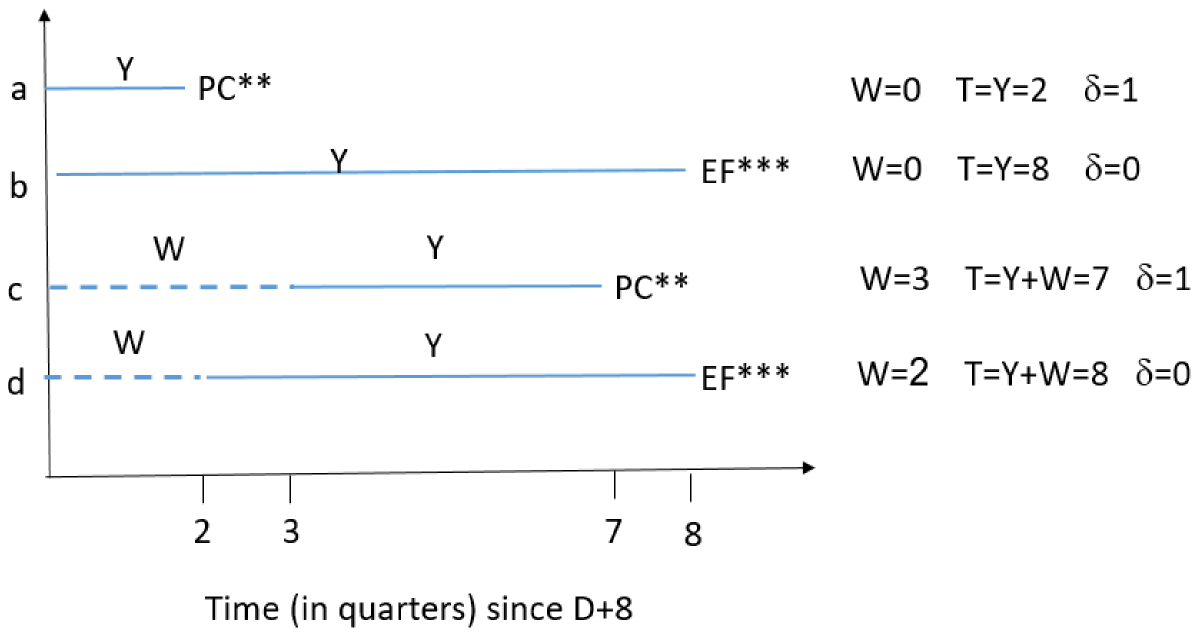

*Adapted from Lamarca et al[1]
** Pancreatic Cancer
*** End of Follow-up

Patient a was diagnosed with diabetes at the 1st quarter of 2008 (j=25) and was diagnosed with pancreatic cancer at the 2nd quarter of 2010 (j=34).

D=25 ; D+8=33 ; F=34 ; W=0 ; Y=34-33+1=2 ; T=2 ; $\delta = 1$

Patient b was diagnosed with diabetes at the 3rd quarter of 2008 (j=27) and died on the 2nd quarter of 2012 (j=42)

D=27 ; D+8=35 ; F=42 ; W=0 ; Y=42-35+1=8 ; T=8 ; $\delta = 0$

Patient c was diagnosed with diabetes at the 4th quarter of 2006 (j=20) and was diagnosed with pancreatic cancer on the 2nd quarter of 2010 (j=34)

D=20; D+8=28; F=34; W=31-28=3; Y=34-31+1=4; T=7; $\delta = 1$

Patient d was diagnosed with diabetes at the 1st quarter of 2007 (j=21) and reached the age of 90 at the 4th quarter of 2010 (j=36)

D=21; D+8=29; F=36; W=31-29=2; Y=36-31+1=6; T=8; $\delta = 0$

© 2024 Dankner R et al. *JAMA Network Open.*

eFigure 2. Directed Acyclic Graph (DAG) for the Causal Association Between Specified GLM (either GLP1-RA or Basal Insulin) and Pancreatic Cancer

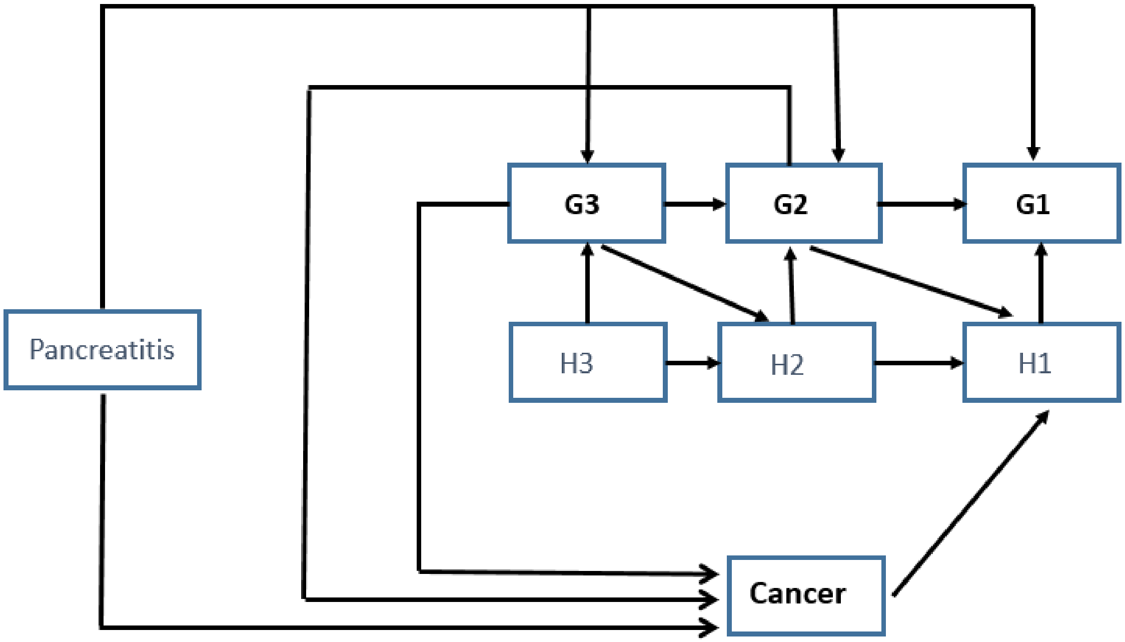

G3 – Average DDD of specified GLM 5th-7th years back

G2 – Average DDD of specified GLM 2nd-4th years back

G1 – Average DDD of specified GLM in previous year

H3 – Average HbA1c level 5th-7th year back

H2 – Average HbA1c level 2nd-4th year back

H1 – Average HbA1c level in previous year

Pancreatitis – Pancreatitis diagnosed before initiation of the specified GLM (either GLP-1RA or basal insulin).

The causal diagram above presents the causal paths between specified GLM (either GLP-1RA or basal insulin) average DDD use in the three non-overlapping time-windows (G1 for previous year, G2 for 2nd-4th years back, G3 for 5th-7th years back) and pancreatic cancer in the current 3-month period. In the diagram, it is assumed that reverse causation occurs only one year before the cancer (see arrow from Cancer to H1). It is also assumed that there is no effect of G1 on the cancer. The nature of the reverse causation of the cancer on G1 is through the HBA1c level in the previous year (denoted H1). If one is interested in estimating the effect of the specified GLM in the second or the third time-windows (G2 or G3) on cancer, then the diagram shows that the variables H1 and G1 are colliders[6] (i.e. variables that are influenced by predictors and outcome), and should not be entered into the model. Furthermore, it is not thought that HbA1c levels in the second or the third time-windows (H2 or H3) are related to cancer, and therefore they do not confound the specified GLM – cancer association and should also be omitted from the model.

The diagram shows that Pancreatitis diagnosed before specified GLM use, potentially influences the GLM use in all future time-windows. Furthermore, it is related to the cancer, so it has a confounding effect and should be adjusted for in the model.

© 2024 Dankner R et al. *JAMA Network Open.*

eAppendix 4. New-User and Prevalent New-User Designs

New-User Design

Our goal was to compare GLP-1RA users to basal insulin users with regards to pancreatic cancer incidence. We aimed to match cases (new GLP-1RA users who had not previously taken basal insulin) to comparator controls (new basal insulin users who had not previously taken GLP-1RA). Such an incident new user cohort design[7,8], when cohorts are defined by new users of the drugs under study (treatment-naïve users), for comparing head-to-head comparisons between two drugs, is often used in pharmacoepidemiology. Because GLP-1RA was introduced into Israel only in 2009, we matched these new users only with new-users of basal insulin who had started basal insulin only from 2009 onwards, even though basal insulin has been in use in Israel since the start of our database in 2002.

Prevalent New-User Design

Sometimes there is a substantial proportion of users of the new drug who have switched from the comparator old drug, and who therefore cannot be included in a new-user design. This can occur when the comparator drug was introduced much earlier than the new drug. This happened in our case, since basal insulin was already in use in 2002, the starting date for our database, and the new drug GLP-1RA became available in Israel only in 2009. Thus many users of the new drug had already received basal insulin before they switched to or added GLP-1RA. These accounted for approximately half of those who used GLP-1RA. The Prevalent New-User (PNU) design extends the active comparator new user design by allowing for the inclusion of initiators of the study drug who were previously on a comparator treatment[7-9]. The PNU design combines the new users who are eligible for the new-user design and comparator controls (called incident new users) together with the set of new users who have been previously exposed to the comparator drug (called prevalent new users) and a separately chosen set of matched comparator controls.

We conducted a new-user design analysis and a PNU (all users) analysis as sensitivity analyses.

Matching

First, we identified the cohort of incident new users of the two drugs (incident new users), where study cohort entry was defined as the calendar quarter since the start of 2009 (start of follow up) of the first initiation of GLP-1RA or basal insulin. We also identified the cohort of prevalent new users who used basal insulin but switched to or added GLP-1RA. For these, the study cohort entry was defined as the calendar quarter when the case switched to or added GLP-1RA. Next, we created exposure sets of GLP-1RA (cases) and basal insulin users (controls) that were defined by the new-user type (incident vs. prevalent), study cohort entry, and deciles of cumulative basal insulin exposure prior to study cohort entry (irrelevant for the incident new-users). Eligibility for an exposure set also required not having been diagnosed previously with pancreatic cancer and being under 90 years old. Exposure sets generally included more basal insulin users than GLP-1RA users. After creating the exposure sets, separate conditional logistic regressions for incident type users and for prevalent users, were used for constructing time conditional propensity scores (TCPS) for initiating or switching/adding GLP-1RA respectively[9]. The covariates of the model included diabetes duration, number of previous glucose-lowering medications, age, sex, socio-economic status (SES), ethnicity, body mass index, HbA1c, and pancreatitis before initiation of drug. Since running these models on the full collection of exposure sets was prohibitively time consuming, we randomly sampled 100 GLP-1RA and 100 basal insulin users from each exposure set to estimate the parameters of the TCPS model. Then, based on the estimated coefficients, we calculated a TCPS for all subjects in each exposure set. Finally, a comparator control was selected for each GLP-1RA user using 1:1 matching with replacement[10], which was done in each exposure set separately, using PSMATCH Procedure (SAS 9.4) with the Greedy

© 2024 Dankner R et al. *JAMA Network Open.*

neighbor matching, with a caliper restriction equal to 0.5 of the SD of the logit of the propensity score.

A positivity check on the matching procedure was done using the “Region=treated” option in PSMATCH, which selects controls whose propensity scores lies in the region of propensity scores of cases.

For the 14,887 incident new-users of GLP-1RA (comprising the new-users design), matching was achieved without replacement, but for the 32,115 prevalent new-users of GLP-1RA, 6,518 required a matched basal insulin user that had already been used for another GLP-1RA user.

Baseline characteristics in the two drug groups after matching are shown in eTable 3 (next page). It may be seen that while matching was fairly successful in creating balance between the two treatment groups, minor imbalances remain, especially in BMI, HbA1c level and history of pancreatitis. For this reason, in our Cox model comparison of pancreatic cancer incidence in the two treatment groups, we adjusted for those three baseline characteristics.

Outcome model

To estimate differences in the risk for pancreatic cancer of GLP-1RA and basal insulin users, marginal Cox models[11] were used, with a robust sandwich covariance matrix estimate, to account for the re-used controls arising from the matching with replacement. Because the two treatment groups were incompletely balanced by the matching procedure (see eTable 3), we adjusted the comparison by introducing covariates for baseline body mass index, baseline HbA1c level and previous pancreatitis.

Since our time-data were grouped into 3-month intervals (quarters), start of follow-up was taken to be the quarter following the study cohort entry. Patients who were diagnosed with pancreatic cancer in the same quarter as the study cohort entry were thus excluded. There were 17 such patients in the new-users design (all in the basal insulin group) and 23 in the prevalent new-users design (22 in the basal insulin group and 1 in the GLP-1RA group).

The Cox analyses were conducted according to the intent-to-treat principle: a patient was retained in his/her original treatment group regardless of later changes in the treatment schedule such as a GLP-1RA user later stopping use of GLP-1RA or adding basal insulin.

Finally, to explore and mitigate the effects of reverse causation, we also compared pancreatic cancer incidence between GLP-1RA and basal insulin users using a delay in start of follow-up of one year and of four years. As explained in the main text, we emphasize the results of the analysis that uses a delay of four years from treatment initiation, since this analysis provides the most protection against the bias of reverse causation.

Estimated hazard ratios of pancreatic cancer among GLP-1RA users compared to basal insulin users, derived from the Cox analyses described above, are shown in eTable 4 (page 11).

© 2024 Dankner R et al. *JAMA Network Open.*

eTable 3. Baseline Characteristics by Treatment Group for the Incident New-Users and Prevalent New-Users Design

Baseline characteristic	Incident new-users design		Prevalent new-users design	
	GLP-1RA (N=14,887)	Basal insulin (N=14,887)	GLP-1RA (N=47,002)	Basal insulin[a] (N=47,002)
Age at entry, mean (SD)	59.8 (9.7)	60.1 (10.5)	60.3 (9.8)	60.1 (10.3)
Diabetes duration(years), mean (SD)	9.4 (4.0)	9.3 (4.3)	10.5 (4.2)	10.5 (4.5)
Sex, %				
Male	47.4	49.8	46.6	47.1
Female	52.6	50.2	53.4	52.9
BMI kg/m^2, %				
<20	0.1	0.1	0.1	0.1
[20,25)	0.9	1.0	1.0	1.2
[25,30)	14.1	15.6	16.2	18.4
[30,35)	40.0	43.4	40.5	42.2
35+	43.5	38.0	40.9	37.0
Missing	1.5	1.8	1.2	1.2
Hba1C, %				
<6.5	4.0	5.6	3.1	3.2
[6.5,7.5)	17.6	17.4	14.1	12.7
[7.5,8.5)	33.3	28.7	30.2	25.1
8.5+	43.6	46.6	51.4	57.6
missing	1.5	1.8	1.2	1.4
Ethnic origin, %				
Ashkenazi Jewish	31.0	27.5	27.9	23.8
Ethiopian and Central African Jewish	0.5	0.7	26.1	30.7
Israeli Arab	21.2	24.2	0.6	0.8
Israeli Born Jewish	23.1	21.4	2.3	2.1
Sephardic Jewish	22.0	24.3	21.6	23.0
Yemenite Jewish	2.2	2.0	21.5	19.7
Socioeconomic status (SES), %				
Low	57.4	62.3	61.0	65.9
Medium	39.2	34.8	35.8	31.2
High	3.3	2.8	3.1	2.9
Missing	0.1	0.1	0.1	0.1
Number of previous GLMs, %				
0-1	20.8	23.0	18.8	18.0
2	38.3	37.8	39.2	39.4
3+	40.9	39.2	42.0	42.7
History of pancreatitis, %	1.0	1.6	1.3	1.8

[a] Owing to using matching with replacement, some basal insulin patients are counted more than once, although each time with possibly different baseline values for the characteristics that are time-dependent

© 2024 Dankner R et al. *JAMA Network Open.*

eTable 4. Estimated Hazard Ratios for the Association of GLP-1RA Exposure vs Basal Insulin Exposure With Pancreatic Cancer

Period of follow-up	New-Users Design HR (95% CI)[a]	Prevalent New-Users Design HR (95% CI)[a]
From drug initiation	0.67 (0.41, 1.07)	1.03 (0.72, 1.47)
Omitting the 1st year after drug initiation	0.84 (0.46, 1.52)	1.07 (0.69, 1.66)
Omitting the first 4 years after drug initiation	0.52 (0.19, 1.41)	0.75 (0.37, 1.53)

[a]adjusted for baseline BMI, baseline Hba1C, and history of pancreatitis

© 2024 Dankner R et al. *JAMA Network Open.*

eReferences.

1. Lamarca R, Alonso J, Gómez G, Muñoz A. Left-truncated data with age as time scale: an alternative for survival analysis in the elderly population. J Gerontol A Biol Sci Med Sci. 1998 Sep;53(5):M337-43. doi: 10.1093/gerona/53a.5.m337. PMID: 9754138.

2. Dankner R, Agay N, Olmer L, et al. Metformin Treatment and Cancer Risk: Cox Regression Analysis, With Time-Dependent Covariates, of 320,000 Persons With Incident Diabetes Mellitus. Am J Epidemiol. 2019 Oct 1;188(10):1794-1800. doi: 10.1093/aje/kwz157. PMID: 31269196; PMCID: PMC6768811.

3. Andersen PK, Gill RD. Cox's Regression Model for Counting Processes: A Large Sample Study. Ann. Statist. 1982; 10(4):1100 – 1120. https://doi.org/10.1214/aos/1176345976.

4. SAS Institute Inc. Counting Process Style of Input, In the PHREG Procedure, SAS/STAT® 9.2 User's Guide, Second Edition, Cary, NC: SAS Institute Inc. (2009).

5. Powell TM, Bagnell ME. Your "Survival" Guide to Using Time-dependent Covariates. SAS Global Forum 2012: Pharma and Health Care Providers. Paper 168-2012. SAS Institute Inc.

6. Ari ML, Greenland S. Causal Directed Acyclic Graphs. J Am Med Assoc 2022; 327(11):1083-1084.

7. Suissa S, Moodie EE, Dell'Aniello S. Prevalent new-user cohort designs for comparative drug effect studies by time-conditional propensity scores. Pharmacoepidemiology and Drug Safety 2017; 26(4):459-468.

8. Tazare J, Gibbons, DC, Bokern M, Williamson EJ, Gillespie IA, Cunnington M, Logie J, Douglas IJ. Prevalent new user designs: a literature review of current implementation practice. Pharmacoepidemiology and Drug Safety 2023; 32(11):1252-1260.

9. Yu OHY, Dell'Aniello S, Shah BR, Brunetti VC, Daigle JM, Fralick M, Douros A, Hu N, Alessi-Severin S, Fisher A, Bugden SC, Ronksley PE, Filion KB, Ems P, Lix LM. Sodium–glucose cotransporter 2 inhibitors and the risk of below-knee amputation: A multicenter observational study. Diabetes Care 2020; 43(10):2444-2452.

10. Austin PC, Cafri G. Variance estimation when using propensity-score matching with replacement with survival or time-to-event outcomes. Statistics in Medicine 2020; 39(11):1623-1640.

11. Lee EW, Wei LJ, Amato DA. (1992). "Cox-Type Regression Analysis for Large Numbers of Small Groups of Correlated Failure Time Observations." In Survival Analysis: State of the Art, edited by J. P. Klein and P. K. Goel, 237–247. Dordrecht, Netherlands: Kluwer Academic.

© 2024 Dankner R et al. *JAMA Network Open.*
