# Supplementary material for: Glucagon-Like Peptide-1 Receptor Agonists and Pancreatic Cancer Risk in Patients With Type 2 Diabetes
Source: JAMA Netw Open. 2024 Jan 4;7(1):e2350408. doi: 10.1001/jamanetworkopen.2023.50408 (PMC10767614; doi:10.1001/jamanetworkopen.2023.50408)
Supplement: Supplement 2. — Data Sharing Statement [file jamanetwopen-e2350408-s002.pdf]

Data Sharing Statement

Dankner. Glucagon-Like Peptide-1 Receptor Agonist and Pancreatic Cancer Risk in Patients With Type 2 Diabetes. *JAMA Netw Open*. Published January 04, 2024. doi:10.1001/jamanetworkopen.2023.50408

Data
Data available: No

Additional Information
Explanation for why data not available: The data that support the findings of this study are available from the Clalit Healthcare Services health maintenance organization (HMO), the Clalit Research Institute, Innovation Division, 101 Arlozorov St., Tel Aviv, Israel
